# Gastric Outlet Obstruction Caused by Foley Catheter: A Complication when Substituting for Commercial Gastrostomy Tubes

**DOI:** 10.5811/cpcem.2017.10.35930

**Published:** 2018-01-09

**Authors:** Sawlar Vu, Amanda B. Lewis, Brooks Moore

**Affiliations:** * Emory University, Department of Emergency Medicine, Atlanta, Georgia; † Emory University, School of Medicine, Atlanta, Georgia; ‡ Grady Memorial Hospital, Emergency Care Center, Atlanta, Georgia

## Abstract

The technique of using percutaneous endoscopic gastrostomy (PEG) for long-term enteral feeding is well established and commonly used. While the technique is relatively safe and simple, the gastrostomy tube itself may deteriorate or malfunction, requiring a replacement tube. We present a case of a 58-year-old woman who was found to have gastric outlet obstruction from the inflated balloon of a Foley catheter being used as a replacement for her PEG tube. This case illustrates a potential complication of using a Foley catheter in place of commercially available gastrostomy tubes.

## INTRODUCTION

The technique of using percutaneous endoscopic gastrostomy (PEG) for long-term enteral feeding is well established and commonly used. While the technique is relatively safe and simple, the gastrostomy tube itself may deteriorate or malfunction, requiring a replacement tube. The emergency physician is often the first provider to encounter a patient after PEG tube malfunction. A common practice, appearing in the literature as early as 1992, has been to temporarily replace the malfunctioning or displaced PEG tube with a Foley catheter to avoid stoma closure or narrowing.^1^ Foley catheters may be used if a commercial gastrostomy tube is not available or if a cheaper, temporary alternative is desired.^2^,^3^ While a recent large pediatric trial showed their successful utilization without severe complications, multiple case reports suggest the procedure is not without risk.^4^ This case report presents a patient who was initially identified as having an upper gastrointestinal bleed and was later found to have gastric outlet obstruction as a result of a migrating Foley catheter serving as a PEG replacement.

## CASE REPORT

A 58-year-old African-American female with a history of traumatic brain injury presented to the emergency department (ED) from her nursing home after experiencing several episodes of coffee-ground emesis that started on the day of presentation. She was noted to have three additional episodes of coffee-ground emesis mixed with maroon-colored clots in the ED but did not appear to be in distress. Past surgical history included the placement of a 25 French (Fr) PEG tube approximately one year prior. The PEG had been replaced by a Foley catheter at some point, but there was no available associated documentation.

On physical examination, she was normotensive (133/83 millimeters of mercury), tachycardic (109 beats per minute), and tachypneic with mildly increased work of breathing. She was afebrile and her oxygen saturation was greater than 95%. Her abdomen was distended, firm, and tender to palpation throughout. She demonstrated some guarding with no rebound. Normal bowel sounds were present and no hepatosplenomegaly was noted. Rectal exam revealed brown stool that was guaiac negative. The feeding tube site was clean and without erythema, with a Foley catheter entering the site secured by fenestrated gauze.

**Image 1:**
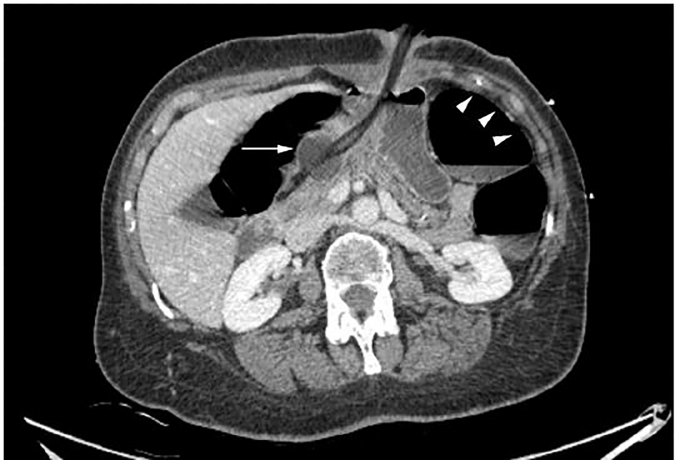
Computed tomography of the abdomen and pelvis, axial view, demonstrating the Foley catheter with balloon in the gastric antrum (arrow), with resulting gastric outlet obstruction and colonic distension (arrowheads).

**Image 2:**
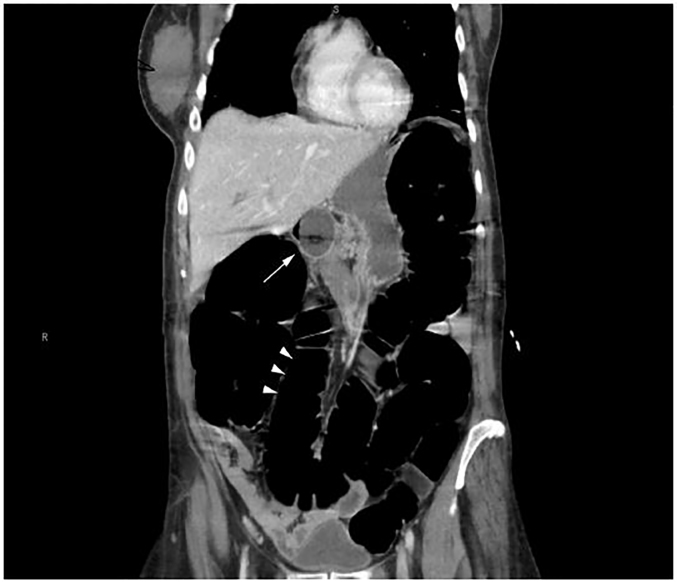
Computed tomography of the abdomen and pelvis, coronal view, demonstrating the Foley catheter with balloon in the gastric antrum (arrow), with resulting gastric outlet obstruction and colonic distension (arrowheads).

The patient was resuscitated following institutional treatment guidelines for sepsis. Laboratory data were within normal parameters except for a lactic acid of 4.2 mmol/L. In addition, there was some concern the patient may have had gastrointestinal obstruction or perforation. The patient was taken for computed tomography (CT) of the abdomen and pelvis with intravenous (IV) contrast. Imaging revealed the Foley catheter traveling through the PEG tract with the balloon resting in the gastric antrum. The stomach was filled with fluid, the small bowel was unremarkable, and the colon was diffusely distended with air-fluid levels and no transition zone (Image 1 and 2).

The Foley balloon was deflated, pulled back, and re-inflated. Following retraction of the Foley catheter, the patient did not experience any further episodes of emesis. The patient was admitted to the inpatient medicine service for urgent endoscopy and observation. The next day the patient underwent endoscopy during which the Foley catheter was replaced with a 20 Fr PEG tube. The lactic acidosis resolved with IV hydration, and no source of bleeding was identified.

## DISCUSSION

PEG is a widely performed procedure and remains the procedure of choice for providing enteral access for nutritional support for adults. There are several risks and complications that should be considered before tube placement. In published meta-analyses and case series, PEG tube placement is associated with notable patient morbidity (9% – 17%) and mortality (0.53%).^5^ Major complications after tube placement occur in 1–3% of cases and include aspiration pneumonitis, peritonitis, hemorrhage, tube migration, fistula, wound infection and necrotizing fasciitis, tube leakage, tube blockage, and inadvertent removal of PEG.^5^

Many professional organizations have guidelines for the use of PEG tubes, but almost none address the use of replacement Foley catheters. In 2008 the Canadian Agency for Drugs and Technologies in Health and their Health Technology Inquiry Service conducted a review of the guidelines and clinical evidence for using Foley catheters for gastrostomy or jejunostomy feeding tubes. They concluded that “no relevant health technology assessments, systematic reviews, meta-analyses, or randomized controlled trials were identified examining clinical effectiveness or potential risk or harms of using Foley catheters versus conventional gastrostomy or jejunostomy feeding tubes in adult inpatients requiring enteral feeding.”^6^ Other leading organizations, including the American Society for Parenteral and Enteral Nutrition, the American Society for Gastrointestinal Endoscopy, and the American Gastroenterological Association, fail to address the specific use of Foley catheters as replacement tubing.^6^ The Wound, Ostomy and Continence Nurses Society™ is the only organization that clearly discourages the use of Foley catheters in enteral feeding and highlights that the Foley catheter is associated with higher complication rates than standard commercially available tubing.^6^

CPC-EM Capsule

What do we already know about this clinical entity?

Foley catheters are frequently used by emergency medicine providers as placeholders or temporary replacements for damaged or displaced percutaneous endoscopic gastrostomy (PEG) tubes

What makes this presentation of disease reportable?

This is a unique complication of replacement Foley catheter use, presenting as a gastrointestinal bleed secondary to gastric outlet obstruction.

What is the major learning point?

While “cheap” and widely available, Foley catheters used in lieu of commercial PEG tubes can be associated with varied complications. Consider using a replacement with a bolster.

How might this improve emergency medicine practice?

Foley catheter complication should be considered in evaluation of the appropriate patient who presents with signs of bowel obstruction and GI bleed.

There are several other reviews and reports that indicate complications associated with the specific use of Foley catheters in enteral feeding. In a study comparing three different types of feeding tube replacements, the Foley catheter was associated with increased incidence of breakage, leakage, and migration.^7^ There are also multiple case reports of pancreatitis and intussusception from Foley catheters used as feeding tubes, often caused by catheter migration.^8^–^10^ Peristalsis-induced migration has also been implicated in other cases of gastric outlet obstruction, often involving the duodenum and other small bowel.^11^^–18^

Some researchers have indicated that complications may be minimized if specific precautions are taken to secure Foley catheters. One prospective randomized trial that compared the Foley catheter as a replacement for commercial gastrostomy tubes in 46 patients found the Foley to function well and showed a low incidence of balloon rupture and no cases of bowel obstruction in the Foley group.^3^ Study personnel specifically secured the tubes with self-made retention rings. Other studies suggest that user error, not the catheter itself, is accountable for complications.^19^

## CONCLUSION

Emergency physicians should be aware of the multiple complications of Foley catheters being used as replacements for PEG tubes. We hope that this case study contributes to the growing body of evidence of Foley balloon-associated gastrointestinal obstruction in PEG-dependent patients. Awareness of this complication enables the emergency physician to potentially treat this condition completely and immediately and avoid serious subsequent complications.
